# Oral Presentations 8

**DOI:** 10.1038/sj.bjc.6601933

**Published:** 2004-06-22


### ORAL PRESENTATIONS 8

### 8.1

### CHANGES IN DYNAMIC CONTRAST-ENHANCED MRI AS AN EARLY PREDICTOR OF RESPONSE TO NEOADJUVANT CHEMOTHERAPY IN PRIMARY BREAST CANCER

MW Ah-See [1], A Makris [1], NJ Taylor [1], M Harrison [1], PJ Ostler [1], PI Richman [1], JJ Stirling [1], RJ Burcombe [1], MO Leach [2], MR Pittam [3], D Ravichandran [3], JA D'Arcy [2], AR Padhani [1]
[1] Mount Vernon Hospital, Northwood, United Kingdom, [2] Royal Marsden Hospital, Sutton, United Kingdom, [3] Luton & Dunstable Hospital, Luton, United Kingdom

**Background:** The ability to identify early during treatment those women with primary breast cancer (PBC) who will fail to respond to neoadjuvant chemotherapy (NAC) will enable the use of alternative therapies that may be more effective. Here, we assess the role of dynamic contrast-enhanced MRI (DCE-MRI) to identify the non-responders. **Methods**: 28 patients with PBC (median age 46 years; range 29-70) were imaged prior to & following 2 cycles of 5-fluorouracil, epirubicin & cyclophosphamide NAC. DCE-MRI was performed using gadolinium & parametric images were calculated reflecting tissue microvessel permeability, leakage space & perfusion ($K^{trans}$, $v_e$, $k_{ep}$, MaxGd, rBV, rBF & MTT). Median & 5-95th centile values for each parameter were derived from whole tumour regions of interest. Pre-treatment parameter values & treatment changes were correlated with clinical & pathological response following 6 cycles of NAC using the Mann-Whitney U-test. A cohort of 9 patients was imaged twice prior to therapy to calculate the repeatability statistic for each parameter & hence determine the ability of DCE-MRI to predict pathological non-response on a patient-by-patient basis.
**Results:** Clinically there were 19 responders & 9 non-responders; pathologically there were 11 responders & 17 non-responders. Pre-treatment parameter values & change in tumour size did not predict for response. Group analysis showed that changes in median $K^{trans}$, $k_{ep}$, rBV & rBF correlated with both final clinical & pathological response to NAC ($p<0.01$) as did changes in 5-95$^{th}$ centile range for these parameters ($p<0.05$). Application of the repeatability statistic revealed change in median $K^{trans}$ to be the best predictor of pathological non-response in individuals (repeatability range -49 to 97%) correctly predicting pathological non-response in all 17 patients (100%) & response in 6/11 patients (55%). **Conclusion**: Patients who are destined to fail to respond to NAC can be identified from early changes in DCE-MRI parameters that reflect on microvessel perfusion & permeability.

### 8.2

### UNEXPECTED UPREGULATION OF MHC CLASS I MOLECULE IN POOR PROGNOSIS BREAST CANCERS

Z Madjd , I Spendlove , SE Pinder , J Ronan , EC Paish , LG Durrant
University of Nottingham, Nottingham, United Kingdom

Background: Tumours can be recognised by either CTL or NK cells. CTL recognition depends on expression of MHC class I loaded with peptides from tumour antigens. In contrast, loss of MHC class I would result in NK activation. The aim of this study was to quantify HLA class I expression in relation to breast tumour prognosis. Methods: A large set of samples (439) from patients with primary operable invasive breast cancer with a mean follow up of 87 months were evaluated for the expression of HLA ABC class I antigen, using HC10 (HLA-ABC heavy chain) monoclonal antibody utilising tissue microarrays. Expression was then compared with different prognostic factors and outcome. Results: Normal breast tissue adjacent to tumour demonstrated high levels of expression of class-I antigens. In contrast, 206 out of 439 (47%) of breast carcinomas were considered negative for HLA Class I expression, 45% (197) showed a reduction in the level of expression and only 35 cases (8 %) expressed high levels of HLA class I. We found a direct relationship between patient survival and HLA-negative phenotype ($p<0.05$); as well as a positive association between histological grade ($p<0.001$), distant metastasis ($p<0.05$), Nottingham Prognostic Index ($p<0.001$), tumour type ($p<0.05$) and recurrence ($p<0.05$) and the level of expression of HLA class I.
Conclusion: Our results lead us to conclude that, unexpectedly, in this large series of invasive breast cancers, down-regulation of HLA ABC was more often found in low grade, good prognosis tumours compared to bad prognosis cancers, also that patients with carcinomas exhibiting lower levels of HLA ABC expression achieved longer survival times. These results suggest that loss of MHC class I may result in NK activation resulting a better tumour prognosis. Future studies will attempt to examine the infiltration of NK cells in this series of breast tumours.

### 8.3

### NCOR1 IS FREQUENTLY ELEVATED IN BREAST CANCER CELLS; MOLECULAR AND CLINICAL SIGNIFICANCE

C.M. Banwell [1], S. Vaisanen [2], M. Guy [3], K.W. Colston [3], C. Carlberg [2], M.J. Campbell [1]
[1] Institute of Biomedical Research, University of Birmingham, Birmingham, United Kingdom, [2] Dept. of Biochemistry, University of Kuopio, Kuopio, Finland, [3] Dept. of Cellular and Molecular Medicine, St. George's, London, United Kingdom

The vitamin $D_3$ receptor (VDR) and other nuclear receptors regulate the transcription of antiproliferative target genes such as p21$^{(waf1/cip1)}$ and control proliferation of normal breast epithelial cells. We proposed in cancer cells that altered activity of NCoR1 and other nuclear receptor co-repressors sustains histone deacetylation (HDAC) activity at target gene promoters resulting in insensitivity to these actions. This can be overcome by co-treatments of nuclear receptor ligand (e.g. $1\alpha,25(OH)_2D_3$) plus HDAC inhibitors. Quantitative real time RT-PCR analyses revealed NCoR1 was frequently (13/19) elevated (>2 fold) in breast cancer cell lines compared to non-malignant MCF-12A cells. Similarly in a panel of matched tumour and normal samples we found highest elevation of NCoR1 in both ER+ve (11 out of 20) and ER-ve (7 out of 16) tumours, with a mean 6.2 and 14.2 fold increase respectively. In both cell lines and tumour material levels of SMRT, a related co-repressor, was co-elevated in 10 samples.
To dissect the molecular significance of NCoR1 elevation we have undertaken comprehensive long range scanning (>10 kb upstream of the transcriptional start) of the human p21$^{(waf1/cip1)}$ promoter in MCF-7 cells by designing 26 over-lapping primer pairs combined with formaldehyde cross-linking chromatin immunoprecipitation (ChIP). This has allowed us to capture the spatial and temporal regulation (0-300 mins post treatment with $1\alpha,25(OH)_2D_3$) of both the histone acetylation status and the interactions of NCoR1 and other proteins on the p21$^{(waf1/cip1)}$ promoter. These studies have found the VDR and its heterodimeric partner retinoid X receptor bind to at least five individual regions of the promoter. This study demonstrates that it would be possible to monitor critical changes in the p21$^{(waf1/cip1)}$ promoter in response to multiple cancer-therapeutic agents such as nuclear receptor ligands, HDAC inhibitors, genistein, and aromatase inhibitors.

### 8.4

### ORAL IDARUBICIN AND ORAL CAPECITABINE IN THE TREATMENT OF LOCALLY ADVANCED OR ADVANCED BREAST CANCER – A DOSE FINDING STUDY

F Nussey [1], J Afseth [2], E Murray [2], A Bowman [2], R Leonard [3], D Cameron [2]
[1] University of Edinburgh, Edinburgh, United Kingdom, [2] Edinburgh Cancer Centre, Edinburgh, United Kingdom, [3] South West Wales Cancer Institute, Swansea, United Kingdom

**Introduction.** Anthracyclines and 5 FU are widely used and effective drugs for the treatment of breast cancer. In advanced breast cancer response rates of over 70% can be achieved using a schedule of weekly bolus adriamycin and continuous infusional 5FU. Many patients however prefer oral chemotherapy, so we have developed an oral regimen using idarubicin and the 5FU pro drug capecitabine.
**Materials and Methods.** 30 post menopausal patients were recruited, 17 in the dose finding phase and 13 in the expansion phase. The starting doses were 10mg/m$^2$ idarubicin days 1-3 and capecitabine 750mg/m$^2$ days 1-14, repeated every 21 days. Doses were escalated as follows; 10/750 (n=6), 10/1000 (n=3), 10/1250 (n=5), 12.5/1000 (n=3), with the expansion phase at 10/1000 (n=12). Patients were evaluated for toxicity with each cycle and for response at cycles 3 and 6.
**Results.** The median age of patients was 66 (54 –76), and the mean number of cycles dispensed was 5 (1-12). Two patients had chemo-naive locally advanced breast carcinoma; the remainder received this regimen as first line chemotherapy for metastatic or locally recurrent disease. There were three deaths within 4 weeks of receiving trial medication, 2 attributable to progressive disease and one related to haematological and treatment related toxicity at the highest dose level. The dose limiting toxicity was neutropenia at 12.5/1000. In the dose finding phase there was one complete and 6 partial physician reported responses. In the extension phase there were 2 complete and 2 partial responses in 7 patients confirmed by independent radiological review to give a confirmed response rate of 57%.
**Conclusions.** We believe that we have developed a feasible oral cytotoxic regimen for the treatment of post-menopausal advanced breast carcinoma, with encouraging evidence of disease activity. The 10/1000 combination requires further evaluation in a phase 2/3 setting.
Gabra H, Cameron DA, et al. Br J Cancer. 1996 Dec;74(12):2008-12.

© 2004 Cancer Research UK

### 8.5

### UNCOVERING COMPLEX PATTERNS OF CPG ISLAND METHYLATION ASSOCIATED WITH CLINICAL OUTCOME IN OVARIAN CANCER

J.A Hall [1], J Paul [1], S.H Wei [2], T.H.M Huang [2], R Brown [1]
[1] Centre of Oncology and Applied Pharmacology, Glasgow University, Glasgow, United Kingdom, [2] Department of Molecular Virology, Immunology, and Medical Genetics, The Ohio State University, Columbus, United States

We aim to extract and prioritize information from complex methylation data to aid design and to direct chemotherapy for ovarian cancer. A supervised method of hierarchical clustering (Hastie, T, Tibshirani, R. et al. 2001, Genome Biol, 2, 3.1) was applied to uncover correlations between groups of CpG islands (CGI) and their synergistic interactions and an endpoint related to chemo-responsiveness of ovarian tumours. Groups of CGIs and their interactions may provide more robust indicators of clinical outcome than individual CGIs.
We developed a program to apply tree harvesting to data from Differential Methylation Hybridisation (Wei, S.H., Chen, C.M. et al. 2002 Clin Cancer Res, 8, 2246), a micro-array based method for assessing methylation. Data was collected from 948 CGI in 18 tumour samples with known patient progression free survival (pfs). Hierarchical clustering of the 948 CGI (distance: Pearson's correlation coefficient, method: complete linkage) divided the dataset into (2x948) -1 clusters. All unique average gene profiles of clusters and two-way interactions of profiles with terms already in the regression model were entered into Cox regression analysis using pfs as the outcome measure. Single CGIs were considered alongside groups of CGIs. The best model in terms of fit and predictive ability was selected using a score statistic.
Initial results suggest a single CGI and a group of eight CGIs are associated with pfs ($p = 0.0006$, $p = 0.003$ respectively), on cross validation the single CGI is selected as the best predictor ($p = 0.021$).
Identifying single CGIs and groups of CGI may provide insight into coordinated methylation of multiple CGIs involved in drug resistance, and aid prediction of patient response to chemotherapy but careful validation of results is required to assess the robustness of complex associations.

### 8.6

### CHEMODENSE CISPLATIN AND ORAL ETOPOSIDE TREATMENT IN RELAPSED OVARIAN CANCER

WA Verborg , LR Campbell , MS Highley , EM Rankin
Division of Cancer Medicine, Ninewells Hospital, Dundee, United Kingdom

**Introduction**: The optimal treatment of relapsing or progressive ovarian cancer after initial platinum-based therapy remains a challenge. Treatment of platinum-resistant disease (relapse <6 m) is controversial. A weekly chemodense regimen combining cisplatin and etoposide has reported activity in this group (Van der Burg et al, British Journal of Cancer (2002) 86, 19-25). We retrospectively reviewed the experience with this regimen in our institution. **Methods**: 33 patients with recurrent or progressive bulky stage IIIc/IV ovarian cancer were treated. Cisplatin (50mg/m$^2$) in normal saline was given weekly, weeks 1-3 and 5-7, combined with oral etoposide (50mg daily, days 1-15 and 29-43). Responders and those with stable disease, assessed by Ca125 and imaging, were then commenced on maintenance oral etoposide therapy (50mg daily for 21 days out of 28) for 6-9 cycles, or until disease progression. **Results**: The first 26 patients have now been analysed. All patients had previously received carboplatin and a taxane. The median number of prior regimens was 1 (range 1-3). The platinum-free interval (PFI) was < 6 months in 10 patients, 6-12 months in 8 patients and >12 months in 8 patients. Following induction therapy, the overall radiological response rate (CR+PR) was 64% (95% CI 43-81%), stable disease 36% and CA125 response rate 96% (95% CI 79-99%). The cisplatin/etoposide induction phase was well tolerated. Grade 3/4 toxicity experienced: leucopenia 35%, fatigue 10%, neutropenic fever 15%, nausea and vomiting 5%. A median of 5 cycles (range 1-15) of maintenance etoposide was given and toxicity was minimal. Overall median survival was 16.5 m. Median survival was 10.5 m in the platinum-resistant group, 17.5 m in those with a platinum-free interval of 6-12 m and 18.5 m in those with a platinum-free interval >12 m. Median progression free interval on imaging was 9 m. **Conclusion**: Weekly cisplatin/etoposide, followed by maintenance oral etoposide, is an active and well tolerated regimen in relapsed or progressive ovarian cancer, even in platinum-resistant patients.

### 8.7

### IS 10-YEAR FOLLOW UP OF OVARIAN CANCER WORTHWHILE?

F Azribi , M Mackean , M Stewart , J.F Smyth
Edinburgh Cancer Centre,Western General Hospital, Edinburgh, United Kingdom

**Objective**: To determine long-term survival and predictors of recurrence in patients with ovarian cancer who survived over 5 years.
**Design**: The Database (from January 1984 to December 1998) was reviewed with 372 ovarian cancer patients who had survived relapse free for over 5 years. 30 patients have since relapsed (8.1%). Case notes were examined for prognostic factors and results of relapse.
Results:
Follow up: Median period of follow up 10 years (Max 19.6 yrs).
**Mode of detection:** Of the 30 patients who relapsed, 7 were discovered on routine follow up with a positive yield of follow-up of 0.018%. 12 were referred by GP, 4 were self-referrals, 3 were an incidental finding at another medical specialty, and 4 no available information. 11, 9 and 6 patients relapsed in year 5, 6 and 7 respectively. The median age for relapsed was 56 (range 23-81).
**Survival** 5 patients are still alive post relapse. Median survival post relapse = 19.1 months (95% Confidence interval 13.3 -28.1 months). Positive predictors for relapse at 5 years were advanced stage ($p=0.0001$), serous pathology ($p=0.0001$) and high grade of tumour ($p=0.026$). Negative predictors were age, performance status, CA 125 at presentation, serum albumin, and alkaline phosphatase levels.
**Conclusion:** Ovarian cancer patients who survive over 5 years have excellent long-term survival rates. The predictors for relapse at 5 years are no different from those at initial presentation. Is the annual review at Oncology clinics the best way to detect the relapse, and cost-effective?

© 2004 Cancer Research UK

